# Quality of Pharmaceutical Industry Press Releases Based on Original Research

**DOI:** 10.1371/journal.pone.0002828

**Published:** 2008-07-30

**Authors:** Bindee Kuriya, Elana C. Schneid, Chaim M. Bell

**Affiliations:** 1 Department of Rheumatology, University of Toronto, Toronto, Ontario, Canada; 2 Department of Medicine, St. Michael's Hospital, Toronto, Ontario, Canada; 3 Departments of Medicine and Health Policy Management and Evaluation, The Institute for Clinical Evaluative Sciences, Faculty of Medicine, St. Michael's Hospital, Toronto, Ontario, Canada; Copenhagen University Hospital, Denmark

## Abstract

**Background:**

Press releases are a popular vehicle to disseminate health information to the lay media. While the quality of press releases issued by scientific conferences and medical journals has been questioned, no efforts to assess pharmaceutical industry press releases have been made. Therefore, we sought to systematically examine pharmaceutical company press releases about original research for measures of quality.

**Methodolgy/Principal Findings:**

Press releases issued by the ten top selling, international pharmaceutical companies in the year 2005 were selected for evaluation. A total of 1028 electronic press releases were issued and 235 were based on original research. More than half (59%) reported results presented at a scientific meeting. Twenty-one percent of releases were not explicit about the source of original data. While harms or adverse events were commonly cited (76%), study limitations were rarely noted (6%). Almost one-third (29%) of releases did not quantify study results. Studies presented in abstract form were subsequently published within at least 20 months in 53% of cases.

**Conclusions:**

Pharmaceutical company press releases frequently report basic study details. However, readers should be cautioned by the preliminary nature of the data and lack of identified limitations. Methods to improve the reporting and interpretation of drug company press releases are desirable to prevent misleading media coverage.

## Introduction

The press release was first introduced in 1906 by Ivy Lee, considered the father of modern public relations [1]. Since then, the medical world increasingly relies on the press release to attract media attention and disseminate health information thought to have “news” value to the general public. Press releases are issued routinely by the pharmaceutical industry. These enterprises use this medium to maintain media visibility and for direct communication with shareholders, the medical community, and newsmakers at large.

There is growing interest in news releases from scientific conferences and medical journals. Studies with accompanying press releases are better represented in the lay media and considered more newsworthy by health journalists [2], [3]. Yet, many caution that press releases do not serve as a precise medical reference. They frequently report incomplete information or omit basic study data [4]. In addition, an editorial process to confirm accuracy of the results is usually lacking.

Since the motivation for issuing press releases by profit-driven companies may be different than those from medical journals or scientific conferences, it is important to examine the quality of this information for commercial bias. No attempts to analyze drug company press releases have been made despite descriptions about the poor quality of conference and journal press releases. Thus, our objective was to systematically examine pharmaceutical company press releases of original research for descriptors of study quality. We did not seek to confirm accuracy of the factual content contained within the releases.

## Methods

We identified international pharmaceutical companies based on their 2004 worldwide prescription sales [5]. We selected the top 10 companies, which represent ∼90% of the global pharmaceutical market share [6]. Only those with publicly accessible web sites and available electronic press releases for the year 2005 (fifth-placed Merck & Co. was excluded as study press releases were not publicly available) were selected for study (Appendix S1). We only included English language releases, which referred to original research. Releases about financial updates or organizational policy were excluded.

No standard format to evaluate press releases exists. However, a previous study developed a coding scheme to record quality measures relevant to journal press releases (4 and Woloshin, personal communication, 2005). We modified this tool with what we believe to be important descriptors of press release quality based on other methodological tools used for critical appraisal [7]. These include descriptors such as reporting of study size, study design, study subjects, duration of follow-up and mention of harm or limitations of the research (Appendix S2). We also documented if results were quantified as relative or absolute risks. We performed PubMed and MEDLINE searches until August 2007 (minimum 20 months) to identify subsequent publication of abstracts presented at scientific meetings and categorized them based on their Institute for Scientific Information 2007 impact factor [8]. Journals were classified as “high-impact” if their impact factor was among the top 10 in their relevant category.

All releases were coded by a study author (BK). A random sample of 10% of press releases for each company was coded by another author (ES) to establish the reliability of the coding scheme. Inter-rater reliability using the κ statistic ranged from 0.80 to 1.0 with a mean of 0.90, representing “almost perfect” agreement [9].

## Results

Between January 1, 2005 and December 31, 2005, the 10 companies issued a total of 1028 electronic press releases. Almost a quarter of these (n = 235, 23%) were based on original research but the number varied among companies (range 7–54). The most commonly represented medical disciplines included studies of cardiovascular (20%), oncology (20%) or HIV/AIDS (9%) research.

More than half (59%) of the press releases reported results presented as abstracts at a scientific meeting (Table 1). Some (20%) described studies published in peer-reviewed journals but many (38%) did not provide full reference to the corresponding journal article. The remainder of releases (21%) did not mention the source of original data at all.

**Table 1:** Quality of press releases by study type issued by top selling pharmaceutical companies.

	RCT	Non-RCT	No. (% of total)
**Description**			
**No. (%)**			
Press releases based on original research	158 (67)	77 (33)	235 (100)
Median sentences	46	35	
Study presented at scientific meeting	92 (67)	46 (33)	138 (59)
Study published in medical journal	31 (66)	16 (34)	47 (20)
**Quality Measures**			
**No. (%)**			
Study size reported	137 (72)	54 (28)	191 (81)
Study subjects reported	162 (69)	73 (31)	235 (100)
Follow-up time reported	119 (72)	47 (28)	166 (71)
Study results quantified	118 (72)	46 (28)	164 (71)
Harms/adverse events reported	128 (72)	50 (28)	178 (76)
Limitation noted	10 (77)	3 (23)	13 (6)
Study author quoted	77 (70)	33 (30)	110 (47)
Other funding source cited	69 (70)	30 (30)	99 (42)

RCT, randomized controlled trial; non-RCT includes press releases of uncontrolled studies, controlled but not randomized studies, surveys, diagnostic test studies and unknown study type.

Basic study details were frequently reported. Of those reporting study design, 67% were randomized controlled trials (RCTs), 14% were uncontrolled trials and the remainder (9%) included survey and cohort studies. Study size was frequently recorded. The majority (60%) were medium-sized studies (31–1000 people) involving human subjects (99%). Follow-up time and quantification of study results was included in 71% and more common in press releases of RCTs. When quantified, 27% provided a base rate while 73% presented a ratio measure, most commonly as a relative risk.

Harms or adverse events were cited in about three-quarters (76%) of releases but study limitations (6%) were rarely noted, with the majority (62%) reporting the results as preliminary. Almost one-half of releases (47%) quoted a study author who was usually the principal investigator. The authors' comments typically emphasized the benefit of the intervention but only 10% described limitations of the research. Additional sources of study funding were also reported (42%) and typically involved partnership with another pharmaceutical company.

**Figure 1:**
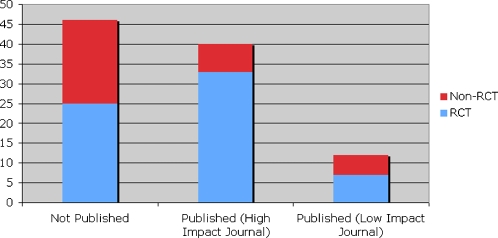
Percentage of scientific meeting abstracts published within 20 months. “High-impact journal” was classified as a journal within the top 10-impact factor rating in the relevant specialty area (e.g. cardiovascular, oncology), general medicine category or research and experimental medicine category. Low-impact journals were not in any of the 3 top lists.

Of the 138 scientific meeting-related press releases, 55 (41%) abstracts, primarily of RCTs, were subsequently published in high-impact journals, 17 (12%) in low-impact journals and 64 (47%) remained unpublished when evaluated at a minimum of 20 months after the scientific conferences (Figure 1).

## Discussion

To our knowledge, this is the first study to systematically appraise pharmaceutical press releases. Our large sample provides useful information about the quality of press release reporting of original research.

Our study shows that industry press releases are generally good at providing basic study details, but may provide incomplete information needed for a reader to gauge a study's clinical implications. Many press releases focused on research presented at scientific conferences, often considered the forefront of current knowledge. However, this information is frequently based on preliminary data without peer-review and may omit limitations such as small study size, uncontrolled study design or short duration of follow up [10], [11]. In turn, this can mislead readers about the validity of results [12]. Furthermore, several releases were not explicit about the source of original data and only a fraction noted any study limitations. A third of releases did not quantify study results, and of those that did, results were presented using a ratio measure - a format known to exaggerate the perceived magnitude of effect [13].

It is not surprising that randomized controlled trials were overrepresented in press releases as these studies have considerable influence on clinical practice. In addition, widespread illnesses such as heart disease, cancer and HIV/AIDS were the most cited medical disciplines. Previous work has shown that medications used to treat chronic disorders are the focus of considerable advertising efforts by pharmaceutical companies and may garner more media attention [14], [15].

We found no other work evaluating press releases from pharmaceutical companies. However, Woloshin et al. examined the quality of press releases issued by medical journals [4]. Similar to our data, they found that releases do not routinely highlight limitations and often use formats that exaggerate study findings.

Our study has several limitations. First, we only examined archived releases for the year 2005 available in electronic format on corporate websites. This led to the exclusion of fifth-ranked Merck & Co. While results may not be generalizable to smaller drug companies, we selected the world's most successful pharmaceutical manufacturers who all have strong media presence and public relations practices. Second, we did not corroborate press release content with subsequent publication, as our aim was to report study characteristics rather than test data accuracy. This approach may be justified as previous work has shown that abstracts often differ from published peer-reviewed results. In a study examining transition of scientific meeting abstracts to full-length journal article, 41% of publications exhibited significant discrepancies when compared to the original abstract data [16]. Third, the findings are predicated on our construct of what constitutes a good quality press release. Our instrument was based on previous work and established guides but results may differ with other assessments tools. Lastly, while we know that journalists frequently rely on press releases as a source of health topic ideas, the association between releases and ensuing coverage in newspaper, radio or television stories was not investigated [17].

Our findings suggest that the quality of pharmaceutical press releases needs improvement. Simple ways of enhancing quality would include referencing original data to confirm press release content, quantifying study results in clinically meaningful ways, and identifying important limitations so that the generalizability of results across different populations and settings can be appreciated.

It is unlikely that the pharmaceutical industry will change its reporting of original research. Thus, journalists and newsmakers should be aware of the shortcomings of press release data and learn to scrutinize this information before adoption into health news for public consumption. Creating a standardized “checklist” of quality indicators is one possible appraisal tool for readers to use.

Future studies should explore the process by which press release content is selected, edited and later distributed. As with all health information, critical review is essential to contextualize its content.

## Supporting Information

Appendix S1 (0.06 MB DOC)

## References

[1] Hiebert RE (1966). Courtier to the crowd: the story of Ivy Lee and the development of public relations..

[2] de Semir V, Ribas C, Revuelta G (1998). Press releases of science journal articles and subsequent newspaper stories on the same topic.. JAMA.

[3] Stryker JE (2002). Reporting medical information: effects of press releases and newsworthiness on medical journal articles' visibility in the news media.. Prev Med.

[4] Woloshin S, Schwartz LM (2002). Press releases- translating research into news.. JAMA.

[5] Scrip 100 - A fresh look at what matters to the pharmaceutical industry.. http://www.pjbpubs.com/uploads/downloads/scrip/download_SCRIP100.htm.

[6] Fortune 500 (2004). How the industries stack up.. Fortune.

[7] Guyatt G, Drummond R, Meade M, Cook D (2002). Users' guides to the medical literature: a manual for evidence-based clinical practice..

[8] ISI Web of Knowledge Journal Citation Reports.. http://portal.isiknowledge.com.myaccess.library.utoronto.ca/portal.cgi?DestApp=JCR&Func=Frame.

[9] Landis RJ, Koch GG (1977). The measurement of observer agreement for categorical data.. Biometrics.

[10] Woloshin S, Schwartz LM (2006). Media reporting on research presented at scientific meetings: more caution needed.. Med J Aust.

[11] Schwartz LM, Woloshin S, Baczek L (2002). Media coverage of scientific meetings: too much, too soon?. JAMA.

[12] Woloshin S, Schwartz LM (2006). What's the rush? The dissemination and adoption of preliminary research results.. J Natl Cancer Inst.

[13] Schwartz LM, Woloshin S (2004). The media matter: a call for straightforward medical reporting.. Ann Intern Med.

[14] Rosenthal MB, Berndt ER, Donohue JM, Frank RG, Epstein AM (2002). Promotion of prescription drugs to consumers.. N Engl J Med.

[15] Woloshin S, Schwartz L, Tremmel J, Welch HG (2001). Direct-to-consumer advertisements for prescription drugs: what are Americans being sold?. Lancet.

[16] Toma M, McAlister F, Bialy L (2006). Transition from meeting abstract to full-length journal article for randomized controlled trials.. JAMA.

[17] Van Trigt AM, de Jong-van den Berg LT, Haaijer-Ruskamp FM, Wiellems J, Tromp TFJ (1994). Journalists and their sources of ideas and information on medicines.. Soc Sci Med.

